# Levosimendan in Acute and Advanced Heart Failure: An Appraisal of the Clinical Database and Evaluation of Its Therapeutic Applications

**DOI:** 10.1097/FJC.0000000000000533

**Published:** 2017-08-15

**Authors:** Johann Altenberger, Finn Gustafsson, Veli-Pekka Harjola, Kristjan Karason, Detlef Kindgen-Milles, Matti Kivikko, Gabriella Malfatto, Zoltán Papp, John Parissis, Piero Pollesello, Gerhard Pölzl, Carsten Tschöpe

**Affiliations:** * Rehabilitationszentrum, Großgmain, Lehrkrankenhaus der Paracelsus Medizinischen Privatuniversität, Salzburg, Austria;; † Cardiology, Rigshospitalet, Copenhagen, Denmark;; ‡ Cardiology Clinic, HUS Meilahti Hospital, Helsinki, Finland;; § Cardiology, Sahlgrenska University Hospital, Gothenburg, Sweden;; ¶ Klinik für Anästhesiologie, Universitätsklinikum Düsseldorf Heinrich-Heine University, Düsseldorf, Germany;; ‖ Critical Care, Orion Pharma, Espoo, Finland;; ** Cardiologia, Ospedale San Luca, Istituto Auxologico Italiano, Milan, Italy;; †† Division of Clinical Physiology, Institute of Cardiology, Faculty of Medicine, University of Debrecen, Debrecen, Hungary;; ‡‡ Second University Cardiology Clinic, Attiko Teaching Hospital, Athens, Greece;; §§ Universitätsklinik für Innere Medizin III Innsbruck, Medizinsche Universität, Innsbruck, Austria;; ¶¶ Department of Cardiology, Universitätsmedizin Berlin, Campus Virchow Klinikum (CVK), Berlin, Germany; and; ‖‖ Berlin Center for Regenerative Therapies (BCRT), Campus Virchow Klinikum (CVK), Berlin, Germany.

**Keywords:** inodilators, inotropes, acute heart failure, advanced heart failure, levosimendan

## Abstract

The use of inotropes for correcting hemodynamic dysfunction in patients with congestive heart failure has been described over many decades. However, negative or insufficient data have been collected regarding the effects of cardiac glycosides, catecholamines, and phosphodiesterase inhibitors on quality of life and survival. More recently, the calcium sensitizer and potassium channel-opener levosimendan has been proposed as a safer inodilator than traditional agents in some heart failure settings, such as advanced heart failure. At the 2017 annual congress of the Heart Failure Association of the European Society of Cardiology (Paris, April 30–May 2), a series of tutorials delivered by lecturers from 8 European countries examined how to use levosimendan safely and effectively in acute and advanced heart failure. The proceedings of those tutorials have been collated in this review to provide an expert perspective on the optimized use of levosimendan in those settings.

## INTRODUCTION

Levosimendan is a first-in-class drug that acts as an inodilator through a tripartite mechanism which involves acting as a calcium sensitizer in cardiomyocytes by increasing the sensitivity of troponin C fibers to ionic calcium and as a vasodilator and cytoprotective agent through the opening of adenosine triphosphate (ATP)-dependent potassium channels on vascular smooth muscle cells and in mitochondria.^1^ Since its introduction at the beginning of the 21st century, levosimendan has been evaluated extensively for the treatment of acute heart failure (AHF) and in a range of other settings characterized by impaired cardiac performance, including cardiac surgery and sepsis.^2^ Among drugs broadly classified as inotropes, levosimendan is the most widely researched agent of the past 20 years.^3^

The hemodynamic effects of levosimendan in heart failure comprise significant, dose-dependent increases in cardiac output (CO) and stroke volume and reductions in pulmonary capillary wedge pressure (PCWP) and pulmonary artery pressure.^4^ These effects are seen early after the initiation of intravenous (i.v.) levosimendan therapy and persist (for up to ≈10 days) after cessation of infusion through the influence of the long-acting active metabolite OR-1896.^1^

Multiple meta-analyses have identified tangible clinical benefits from levosimendan; in particular, it is reported to be the only inotrope associated with improved survival^2,3^ and has also been linked with reduced risk of deterioration of heart failure and the associated likelihood of hospitalization.^5^

Recent interest has been directed toward the use of repeated cycles of i.v. levosimendan to avert acute decompensation and frequent rehospitalization in patients with advanced heart failure (AdvHF) and possibly to enhance health-related quality of life.^6^ Unlike dobutamine, levosimendan is effective in patients who have been treated with beta-blockers, and unlike milrinone, it is not detrimental to patients with AHF of ischemic origin.^7,8^ These qualities add to the distinctiveness of levosimendan among inotropic agents and are reflected in its inclusion in the most recent editions of the European Society of Cardiology (ESC) guidelines for the treatment of heart failure as part of the armamentarium of drugs for the treatment of AHF.^9^

This focused review examines 3 current themes in the use of levosimendan: management of AdvHF, treatment of AHF, and preservation of renal function in heart failure.

## INODILATORS IN AHF

### Guidelines of the European Society of Cardiology

The 2016 ESC guidelines for the management of AHF endorse the use of inotropes for “patients with hypotension [systolic blood pressure (SBP) <90 mm Hg] and/or signs/symptoms of hypoperfusion despite adequate filling status to increase cardiac output, increase blood pressure, improve peripheral perfusion, and maintain end-organ function.” Inotropes are also endorsed “…to reverse the effect of beta-blockade if (that) is thought to be contributing to hypotension and subsequent hypoperfusion.”^9^

Pharmacology considerations and clinical data support the view that levosimendan may be well suited to the needs of patients in those situations.

### Calcium Sensitization Versus Calcium Mobilization

Levosimendan is differentiated profoundly from traditional inotropes by the fact that it is a calcium sensitizer that enhances the response of cardiomyocytes to a given concentration of intracellular ionic calcium.^1^ By contrast, adrenergic stimulants, digoxin, and phosphodiesterase inhibitors are all calcium mobilizers that increase the concentration of ionic-free calcium in cardiomyocytes. Calcium mobilizers therefore expose cardiomyocytes to potentially toxic concentrations of ionic calcium and, inter alia, increase myocardial oxygen consumption; levosimendan has neither effect.

In addition, levosimendan exerts vasodilator and cardioprotective effects through action on potassium-dependent ATP channels on cardiac mitochondria and vascular smooth muscle cells^1^ (Box 1). These are significant ancillary actions in the context of AHF with hypoperfusion, venous congestion and endothelial impairment, and deteriorating end-organ (notably renal) function and likely contribute to the quality of symptom relief achievable with levosimendan.

BOX 1. **Levosimendan acts as an inodilator through a tripartite mechanism**

1. Increasing the sensitivity of cardiomyocyte troponin C fibers to ionic calcium;
2. Opening of adenosine ATP-dependent potassium channels on vascular smooth muscle cells;
3. Opening of adenosine ATP-dependent potassium channels in mitochondria.

In aggregate, these pharmacological differences have a wide-ranging impact on the risk–benefit profile for both types of agent and strongly favor levosimendan. Although calcium mobilizers are associated with tachycardia and arrhythmias, proischemic effects, cardiac hypertrophy, apoptosis and fibrosis, and worse medium- to long-term prognosis, levosimendan enhances CO and systolic and diastolic function, promotes vasodilatation and peripheral perfusion, reduces (PCWP), alleviates symptoms of dyspnea and fatigue, and reduces levels of signifier neurohormones such as brain natriuretic peptide (BNP).^10^

**FIGURE 1.**
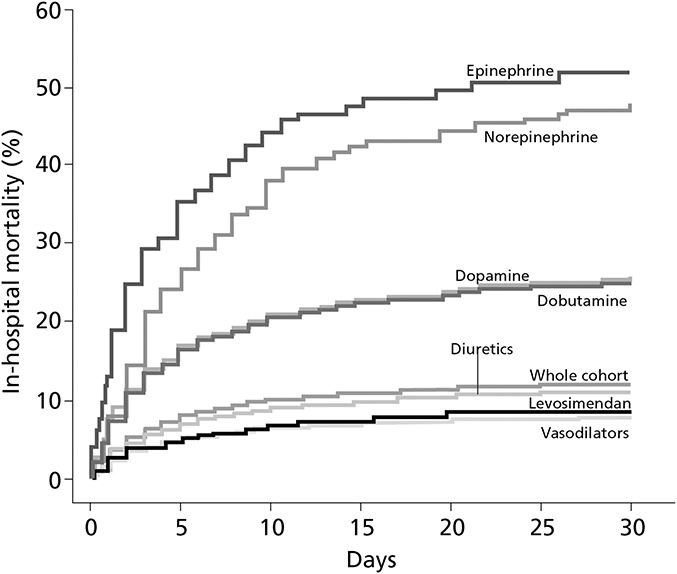
In the ALARM-HF registry, the mixed inodilator profile of levosimendan was associated with a mortality rate substantially lower than that seen with traditional (“calcium-mobilizing”) inotropes, from data by Mebazaa et al.^11^

Perhaps the most significant of all, given the safety concerns attached to inotropes in the 2016 ESC guidelines, is the fact that the mixed inodilator profile of levosimendan is associated in routine clinical practice with a mortality rate lower than that seen with calcium-mobilizing inotropes and much closer to that achieved with vasodilators (Fig. 1).^11^

### Vasodilation, Venous Congestion, and Hypoperfusion

The vasodilator aspects of the clinical pharmacology of levosimendan are likely to be very relevant to the drug's application in low-output states such as AHF and cardiogenic shock. The description of these conditions as “low-output states” is accurate but potentially misleading, given that the central concern in many cases is organ underperfusion. Accordingly, the use of a drug that evokes vasodilatation and augmenting CO may be expected to have a more favorable impact on the prognosis of patients than one that acts only as a cardiac stimulant or which has pressor effects. It may be debated that, in general, a fixation on raising SBP in response to organ hypoperfusion is inappropriate and that at least some patients are being overtreated to maintain blood pressure at the expense of restoring appropriate organ perfusion.

**FIGURE 2.**
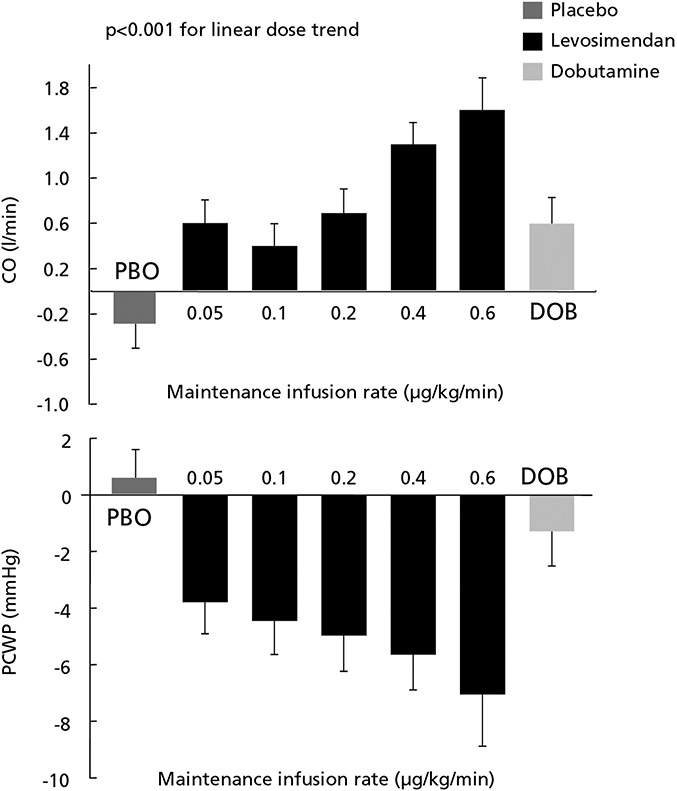
In patients with New York Heart Association class II–IV heart failure of ischemic origin, dosing of levosimendan as a 10-min bolus of 6–24 μg/kg followed by an infusion of 0.05–0.2 μg·kg^−1^·min^−1^ was well tolerated and produced favorable hemodynamic effects, including enhanced CO and stroke volume and reduction in PCWP, derived from Nieminen et al.^10^ PBO, placebo; DOB, dobutamine.

Levosimendan can be used in AHF during episodes of low CO and impaired organ perfusion to:

1. Improve hemodynamics (Fig. 2)^10^ and tissue perfusion;
2. Relieve symptoms of congestion and fatigue^12^;
3. Augment renal blood flow and glomerular filtration rate (GFR) through afferent arteriolar dilatation and increase urine production.

These priorities, and the ability of levosimendan to meet these needs, are acknowledged in the indication of the drug for short-term treatment of acutely decompensated severe chronic heart failure when conventional therapy is not sufficient, and in cases in which inotropic support is considered appropriate (Box 2). Dosage in these situations is guided in part by blood pressure, with bolus omitted or used only if SBP is <100 mm Hg. Meta-analysis of 45 randomized controlled trials in cardiac surgery or cardiology identifies an infusion rate range of 0.05–0.2 μg·kg^−1^·min^−1^, with some indications that lower rates (<0.1 μg·kg^−1^·min^−1^) may confer greater survival advantages over higher doses.^13^

BOX 2. **Expected effects of the use of levosimendan in AHF**

1. Improvement of hemodynamics and tissue perfusion;
2. Relief of symptoms of congestion and fatigue.

Experience from recent large randomized trials indicates that levosimendan can be considered safe in high-risk patients who have been exposed to extensive previous polypharmacy, including beta-blockers.

## LEVOSIMENDAN IN AdvHF

### Background, Rationale, and Theoretical Considerations

AdvHF may be identified in the clinic using the definition of the condition proposed by the ESC (Table 1)^14,15^; it is differentiated from end-stage heart failure by the fact that the cardiac dysfunction and symptoms associated with AdvHF are still potentially reversible, whereas in end-stage heart failure, they are not.

**TABLE 1.**
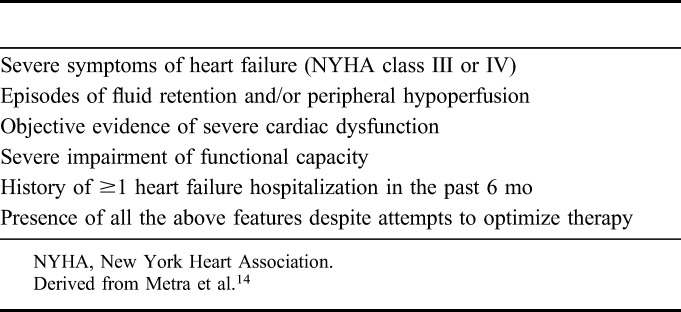
European Society of Cardiology Definition of AdvHF

Within the broad category of AdvHF, however, some patients may be severely or very severely ill but relatively stable, whereas others may be relatively less ill but deteriorating rapidly; in those situations, further medical therapy with i.v. vasoactive drugs may be unnecessary or futile. Cross-referencing of the criteria for Interagency Registry for Mechanically Assisted Circulatory Support^16,17^ grades 4, 5, or 6 with the ESC definition may be used, as proposed by Nieminen et al,^18,19^ to identify the subset of patients with AdvHF most likely to benefit from repeated or intermittent cycles of inodilator therapy.

Patients with AdvHF are on a trajectory which leads eventually either to a definitive intervention through heart transplantation or the installation of a left ventricular assist device, or to a palliative care pathway. In both cases, the goals of therapy include hemodynamic stabilization and preservation of functional capacity, mitigation of symptoms, and preservation of health-related quality of life. Another key goal, particularly in the palliative scenario, is prevention of heart failure–related hospitalization, both as an end in itself and to try to avert the markedly worsened mortality associated with hospitalization, which persists for several months after an admission.^15,17,19–21^

Some commentators have recently suggested that neither New York Heart Association (NYHA) classes IIIb–IV nor INTERMACS levels 4, 5, and 6 are able to correctly identify all high-risk patients nor give a real overview of global health state and the degree of disease of the patients. Heart failure is indeed a complex condition defined by Neubauer^19^ as an “engine out of fuel.” To better identify patients with AdvHF, all the relationships existing between heart, lung, and peripheral organs should be considered and a unique picture representing the clinical global status of the patient that in turn could help to determine treatment.^20^

All in all, in our opinion, levosimendan seems well suited as treatment of AdvHF when given as repeated or intermittent cycles of therapy by virtue of its favorable impact on hemodynamics, its pharmacokinetics, and its persistence of effect (for up to ≈10 days) through its long-acting active metabolite OR-1896. Other relevant qualities include the following:

1. No increase in intracellular calcium concentration or myocardial oxygen demand;
2. No attenuation by the concomitant use of beta-blockers^21,22^;
3. Renal protection (through increase in peripheral organ perfusion)^22,23^;
4. Reduction of natriuretic peptides (considered as biomarkers of a favorable clinical response).

Meta-analyses in cardiological and noncardiological settings have produced encouraging signals; it certainly seems that use of levosimendan in repeated or intermittent cycles is not associated with the distinct increase in mortality reported from use of conventional adrenergic inotropes,^24–26^ but is associated with a reduction of rehospitalization^26^ (Box 3).

BOX 3. **Repetitive Use of Levosimendan in AdvHF**

Observations in the Levo-Rep, LION-Heart, and LAICA randomized clinical trials are indicative of clinical benefits from repetitive-use levosimendan in AdvHF including reduction in NT-pro-BNP levels and trends toward reductions in heart failure readmissions and heart failure–related mortality. Registry data also indicate a reduction in heart failure–related hospitalizations.

Use of levosimendan in repeated or intermittent cycles seems not to be associated with the increase in mortality associated with the use of conventional inotropes.

### Clinical Trial Findings

Three recently concluded, randomized, placebo-controlled, double-blind clinical trials have examined the application of repeated cycles of levosimendan therapy in this setting.

Levo-Rep (NCT01065194),^6^ LION-Heart (NCT01536132),^27^ and LAICA (NCT00988806)^28^ built on observations from earlier open-label studies that suggested benefits from levosimendan in this setting, including improvements in symptoms, hemodynamics and left ventricular ejection fraction, modulation of neurohormonal and immune activation, and, possibly, improvements in survival.^29–32^

As illustrated by their principal inclusion criteria (Table 2), these studies included very similar patient populations. The study protocols for LEVO-Rep and LION-Heart were also very similar. The protocol for LEVO-Rep specified 4 cycles of levosimendan therapy (6-hour administration at 0.2 μg·kg^−1^·min^−1^ every 2 weeks); for LION-Heart, the protocol was extended to include 2 additional cycles of levosimendan therapy in response to intimation from LEVO-Rep that a larger cumulative dose of levosimendan might be needed to fully explore the potential of this intervention. The study dose per cycle was identical in LEVO-Rep and LION-Heart (0.2 μg·kg^−1^·min^−1^ i.v. levosimendan for 6 hours at 2-week intervals); in LAICA, a lower dose but a longer duration of treatment was examined (0.1 μg·kg^−1^·min i.v. levosimendan for 24 hours at 30-day intervals for up to 12 months; median 6 months).

**TABLE 2.**
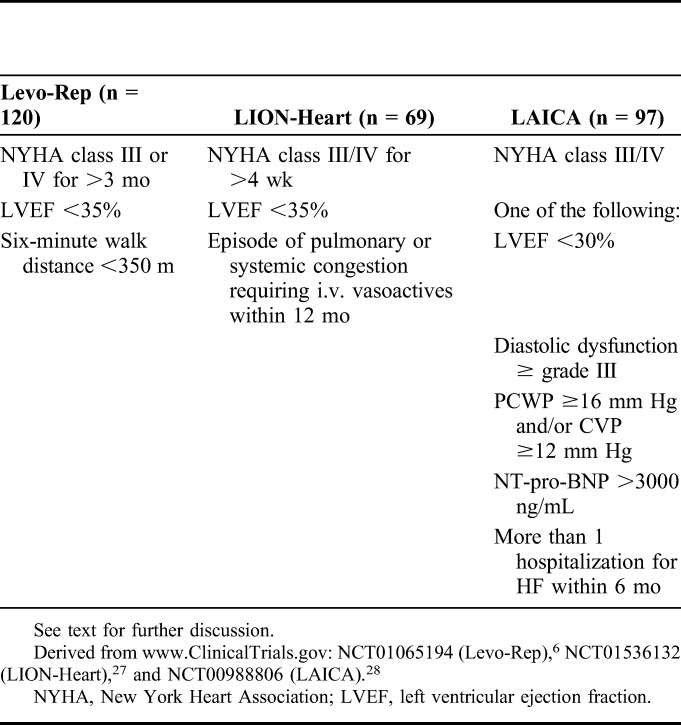
Comparison of Patient Populations in the LEVO-Rep, LION-Heart, and LAICA Trials

LEVO-Rep did not reach its primary endpoint of functional capacity and clinical status, although it might be argued (in retrospect) that this was a highly ambitious outcome. On its secondary endpoints, however, LEVO-Rep documented a significant reduction in N-terminal pro-BNP (NT-pro-BNP) levels at 8 weeks (*P* = 0.006) and an improvement in event-free survival (hazard ratio 0.39, 95% confidence interval 0.15–0.98, *P* = 0.037 by Fisher's exact test). Those findings were recapitulated in LION-Heart, which described a significant benefit from levosimendan on its primary endpoint of NT-pro-BNP levels (*P* < 0.001) and the secondary endpoints of heart failure hospitalization (*P* = 0.002) and all-cause death or heart failure hospitalization (*P* = 0.022). LION-Heart also recorded a significant reduction in the proportion of patients registering a clinically significant decline in heart failure–related quality of life at 6 months (20% vs. 64%; *P* = 0.022). LAICA was inconclusive regarding its primary endpoint of heart failure hospitalization but did reveal an improvement in survival.

In all 3 studies, the safety and tolerability profile of levosimendan compared favorably with that of placebo, and it can be argued that experience in all these studies demonstrated that repetitive application of levosimendan is feasible and safe, even in an outpatient setting.

Only 1 study (LION-Heart) delivered a positive outcome on its primary endpoint, but all these studies demonstrated that repeat-cycle levosimendan reduces NT-pro-BNP levels, and there were repeated and clear demonstrations of trends toward reductions in heart failure readmissions and mortality that are consistent with, and corroborate, the findings of meta-analyses.^2,24,25^

These studies are thus encouraging and strongly suggestive of clinical benefits from repetitive-use levosimendan in AdvHF, but additional larger studies, perhaps in sicker patients, are needed to further elucidate the potential of levosimendan in this setting.

### Clinical Experience and Insights

The hemodynamic effects of levosimendan are well characterized and include enhanced CO and stroke volume and reduction in PCWP.^11^ There are accompanying signs of improved systolic and diastolic ventricular function (eg, Branzi et al^33^).

Hemodynamic effects are central to the use of levosimendan in AdvHF, so much so that the absence of hemodynamic improvement as estimated noninvasively by impedance cardiography predicts 1-year mortality with better sensitivity and specificity than the combination of echocardiographic and BNP criteria.^34^ [Independent predictors of mortality include a <10% increase in the cardiac index or reductions in total peripheral resistance and thoracic fluid content, a persistent restrictive filling pattern (E/E′ ratio >15) and a decline in BNP levels of <30% from baseline.] Positive effects on ventricular function and neurohormonal profile also differentiate levosimendan from agents such as furosemide and may contribute to reduced mortality^35^ and hospitalization rate^36^ in patients with AdvHF.

New insights into the effects of intermittent levosimendan in AdvHF are provided by the RELEVANT-HF registry,^37^ which has compiled data from 185 patients treated at 6 centers in Lombardy, Italy. These patients received repeated levosimendan infusions (0.05–0.2 μg·kg^−1^·min^−1^ without bolus for 24–48 hours at 2–8-week intervals for a minimum of 6 months). Most patients (63%) were treated for relief of symptoms; others were treated as a bridge to transplantation/implantation of a left ventricular assist device (29%) or decision/candidacy (8%).

The primary outcome measure of RELEVANT-HF is the overall duration of hospitalization for heart failure, expressed as the proportion of days spent in hospital during the first 6 months of repeated levosimendan infusion compared with the 6 months before starting treatment. According to that criterion, the use of repeated cycles of parenteral levosimendan was advantageous, reducing the days spent in hospital from 9% to 3%. The ability to deliver a similar scale of benefit from an orally administered regimen would represent a significant advance in the outpatient management of many patients with AdvHF.

**FIGURE 3.**
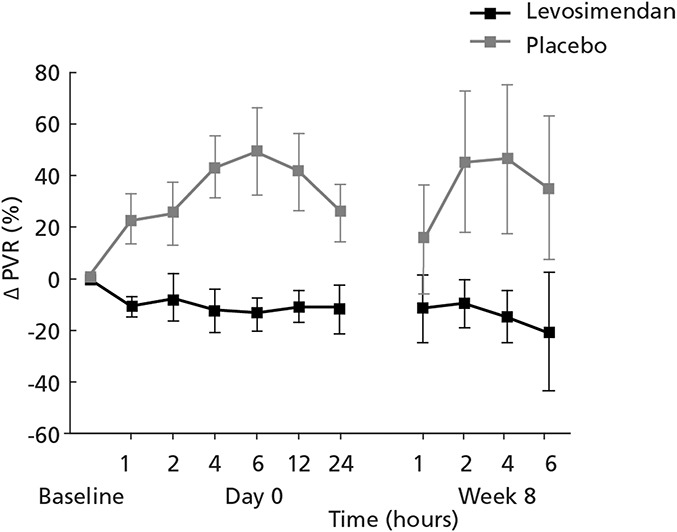
Levosimendan induced sustained reductions in pulmonary vascular resistance (PVR) in patients with pulmonary arterial hypertension, derived from Kleber et al.^38^

The early and sustained reductions in pulmonary vascular resistance achievable with levosimendan suggest that repeat use of this agent may also be beneficial in the management of pulmonary arterial hypertension (Fig. 3).^38^ Published data in this area are limited but encouraging,^38,39^ and further investigations appear warranted.

## EFFECTS OF LEVOSIMENDAN AND OTHER INODILATORS ON RENAL FUNCTION IN ACUTE AND AdvHF

The relation between worsening renal function and deterioration of prognosis in AHF or heart failure decompensation is well established and underlies the strong focus on preservation of renal function in clinical protocols for heart failure management. An important aspect of this situation is, however, often underappreciated: a transient deterioration of estimated GFR, or its surrogate increase in serum creatinine, is not a marker for worse prognosis provided that relief of congestion is achieved. The beneficial effects of drugs targeting the renin–angiotensin–aldosterone system, despite a sometimes marked elevation of serum creatinine, attest to that proposition. By contrast, deterioration of eGFR with persistence of congestion indicates a worse prognosis and an increased mortality. Hence, decongestion is a major target in AHF.

Although it certainly is overly simplistic to profile hemodynamics as the sole cause of end-organ damage, nevertheless, a number of observations highlight the importance of hemodynamic factors in the origins of renal dysfunction in heart failure. In this context, an increase in CO and a reduction in central venous pressure (CVP) are important therapeutic targets. Increasing CO and maintaining an adequate renal perfusion pressure are a quite apparent goal. Regarding CVP, it must be kept in mind that the kidneys are encapsulated organs. Therefore, any increase in pressure within these organs, ie, by edema or backward failure caused by elevated CVP, may reduce the GFR. Increasing CO and reducing CVP by decongestion and fluid removal thus may preserve or restore renal function.

Data from the DOSE study show that the decongestion may require high-dose diuretic strategies and that with successful decongestion improvements in indices of renal function can occur. Effects on “hard” clinical outcomes are, however, less certain.^40^

CO may be augmented by reducing systemic vascular resistance. In situations in which angiotensin-converting enzyme inhibitors and angiotensin receptor blockers are not tolerated (owing to the aforementioned increase in serum creatinine), good effects may be achieved with a hydralazine–isosorbide dinitrate combination. By contrast, the use of low-dose dopamine (so-called “renal” dopamine) is not supported by the results of the DAD-HF trials^41,42^ or the ROSE study.^43^ Similar considerations apply to the use of beta-adrenergic agents. As noted earlier in this commentary application of catecholamines increased mortality in the Alarm-HF registry (Fig. 1).^11^

Levosimendan is one of the few inodilators to have been formally studied for its effects on renal function in heart failure.^44^ The balance of the evidence indicates that it has a favorable and sustained effect, although perhaps not a large one, and that this gain is mediated primarily through hemodynamic actions on both CO and CVP (Box 4).

BOX 4. **Renal Effects of Levosimendan**

The current balance of evidence indicates that levosimendan has a favorable and sustained effect on renal function in AHF, mediated primarily through CO and CVP.

Direct observation of human renal arteries after administration of levosimendan has demonstrated renal arterial vasodilatation and augmented renal blood flow.

In the LIDO study, the sustained improvement in CO observed after levosimendan therapy was associated with a moderate but significant reduction in serum creatinine compared with dobutamine.^22^ A similar effect, also versus dobutamine, was reported by Yilmaz et al,^23^ and a sustained reduction in serum creatinine was demonstrated by Zemljic and colleagues in patients with heart failure on the waiting list for transplantation^22^ and in data from the PORTLAND registry.^45^ No comparable effect was recorded in the large REVIVE I & II trials, although that may be a reflection of the fact that, in those studies, renal data were recorded only as adverse event findings.^46^

Direct observation of human renal arteries after administration of levosimendan has demonstrated that increase in GFR and promotion of diuresis is accompanied by renal arterial vasodilatation and augmented renal blood flow.^47^ Favorable effects on a range of cardiac and vascular echocardiographic indices, biomarker status (including cystatin C), and New York Heart Association heart failure grade and duration of hospitalization have been recorded in an observational study in 96 patients.^48^

These observations are compatible with a hemodynamic model of the effect of levosimendan in which enhancement of CO promotes improved GFR and diuresis, which leads to decongestion and a lower CVP. That in turn, by virtue of a prereducing and afterload-reducing effect, promotes further improvement in both cardiac and renal function. An important aspect of reduced CVP in this context is its favorable impact on right ventricular function. In addition to these clinical findings, observations in a porcine ischemia–reperfusion model of renal failure suggest that, beyond its hemodynamic effects, levosimendan may have additional renal protective qualities arising from antioxidant, antiapoptotic, and cytoprotective actions exercised through the opening of mitochondrial K_ATP_ channels and the generation of intrarenal nitrous oxide.^49^

Levosimendan emerges from these data as a useful addition to the clinical resources available to manage cardiorenal syndrome due to AHF. The published evidence underlines that levosimendan in this setting compares favorable agents such as the vasopressin antagonists (eg, tolvaptan) and adenosine A1 receptor antagonists (eg, rolophylline). New investigational drugs such as istaroxime and ryanodine receptor stabilizers are interesting but of unproven value. Until studies show the efficacy of these new drugs, we suggest a focus on individualized therapy using established drugs after careful consideration of the presenting pathophysiology.

## CONCLUSIONS

Levosimendan occupies a distinctive niche in the management of AHF and AdvHF, producing a significant relief of heart failure symptoms and exerting a variety of well-characterized beneficial effects on hemodynamic, functional, and neurohormonal parameters. Intermittent or repeated courses of levosimendan have been associated with reduced need for heart failure–related hospitalizations and an improved heart-related quality of life. In a number of meta-analyses, a survival benefit from levosimendan compared with conventional inotropes has been reported.^2^ At present, corroboration of that finding in large controlled trials remains elusive.

We must highlight, in fact, that although some trials in the regulatory clinical program (eg, the LIDO trial^21^) showed a superiority of levosimendan versus dobutamine, others did not (eg, the SURVIVE trial^7^). In the literature, there are numerous nonregulatory studies showing a superiority of levosimendan,^13^ but these are usually smaller and often monocentric. The question, again, is which kind of evidence is supported by the large randomized control trials? In the latest 2 decades, many drugs have failed as treatment of AHF as a result of study protocols in which the statistical power required to achieve poorly selected (and overambitious) primary endpoints created the need of large number of patients at a large number of centers.^50^ This need to acquire data from multiple sources introduced variations in pharmacologic and nonpharmacologic measures that had potential to impair the power of statistical insights. As an example pertaining to levosimendan, in the SURVIVE study, some centers applied the study drugs (levosimendan or dobutamine) 1–2 days after randomizations, whereas others waited up to 25 days.^51^ We are therefore not in full agreement that large studies per se produce a definitive answer in fields such as AHF or AdvHF, where signs, symptoms, etiologies, comorbidities, comedications, and center-specific pharmacologic and nonpharmacologic treatment practices are so heterogeneous.

In our opinion, therefore, the hemodynamic benefits and distinguished safety profile in at-risk and clinically unstable patients differentiate levosimendan from conventional inotropes and suggest that its use should be considered more frequently as an alternative to conventional drugs.

Finally, it must be registered that in many studies, including relatively large regulatory clinical trials, levosimendan was administered in addition to standard of care (which means other vasoactive drugs—according to the study centers). It would indeed be intriguing to perform a post hoc analysis of those abundant data to verify if combinations of levosimendan with dobutamine or norepinephrine are beneficial. Until now, only few exploratory studies (eg, Nanas et al^31^) showing a benefit of the combination (levosimendan and dobutamine) have been performed.
